# Short-Term Precision and Repeatability of Radiofrequency Echographic Multi Spectrometry (REMS) on Lumbar Spine and Proximal Femur: An In Vivo Study

**DOI:** 10.3390/jimaging9060118

**Published:** 2023-06-11

**Authors:** Carmelo Messina, Salvatore Gitto, Roberta Colombo, Stefano Fusco, Giada Guagliardo, Mattia Piazza, Jacopo Carlo Poli, Domenico Albano, Luca Maria Sconfienza

**Affiliations:** 1 IRCCS Istituto Ortopedico Galeazzi, Via Cristina Belgioioso, 173, 20157 Milan, Italyluca.sconfienza@unimi.it (L.M.S.); 2 Dipartimento di Scienze Biomediche per la Salute, Università degli Studi di Milano, Via Mangiagalli, 31, 20133 Milan, Italy; 3 Scuola di Specializzazione in Radiodiagnostica, Università degli Studi di Milano, Via Festa del Perdono, 7, 20122 Milan, Italy

**Keywords:** precision, reproducibility, Radiofrequency Echographic Multi Spectrometry (REMS), Bone Mineral Density (BMD), Body Mass Index (BMI)

## Abstract

To determine the short-term intra-operator precision and inter-operator repeatability of radiofrequency echographic multi-spectrometry (REMS) at the lumbar spine (LS) and proximal femur (FEM). All patients underwent an ultrasound scan of the LS and FEM. Both precision and repeatability, expressed as root-mean-square coefficient of variation (RMS-CV) and least significant change (LSC) were obtained using data from two consecutive REMS acquisitions by the same operator or two different operators, respectively. The precision was also assessed in the cohort stratified according to BMI classification. The mean (±SD) age of our subjects was 48.9 ± 6.8 for LS and 48.3 ± 6.1 for FEM. Precision was assessed on 42 subjects at LS and 37 subjects on FEM. Mean (±SD) BMI was 24.71 ± 4.2 for LS and 25.0 ± 4.84 for FEM. Respectively, the intra-operator precision error (RMS-CV) and LSC resulted in 0.47% and 1.29% at the spine and 0.32% and 0.89% at the proximal femur evaluation. The inter-operator variability investigated at the LS yielded an RMS-CV error of 0.55% and LSC of 1.52%, whereas for the FEM, the RMS-CV was 0.51% and the LSC was 1.40%. Similar values were found when subjects were divided into BMI subgroups. REMS technique provides a precise estimation of the US-BMD independent of subjects’ BMI differences.

## 1. Background

Osteoporosis is a systemic skeletal disorder characterized by bone mass loss and deterioration of bone microarchitecture, which results in impairment of bone strength and an increased risk of bone fragility and susceptibility to fragility fractures [1]. Typical sites for osteoporotic fractures are the vertebral spine, proximal femur, and wrist, with hip fractures being associated with significant morbidity in the population [2]. In clinical practice, areal bone mineral density (BMD), expressed as g/cm^2^, is measured by dual-energy X-ray absorptiometry (DXA) and is one of the strongest predictors of fracture risk [3]. The T-score value represents the number of standard deviations that distinguishes the BMD assessed in the patient from the average BMD value in the reference population of young adults. According to the World Health Organization (WHO) diagnostic classification, the status of osteoporosis is defined by a BMD at the hip or lumbar spine that is less than or equal to 2.5 standard deviations (SD) below the average value of the reference population of young adults. Although BMD reflects about 70% of bone strength [4], it resulted in underdiagnosis and undertreatment of osteoporosis since most fragility fractures occur in subjects with BMD out of the osteoporosis classification criteria [5]. This can be explained by assuming that bone strength also depends on microarchitectural changes other than bone density, such as trabecular and cortical thinning, reduced trabecular connectivity, and increased cortical porosity [6]. Additionally, DXA may be prone to possible acquisition and analysis errors, which may lead to inaccurate BMD values [7]. These assumptions have led to the developing of new diagnostic strategies complementary or alternative to DXA. One successful approach for the lumbar spine is the Trabecular Bone Score (TBS), a textural index providing an indirect measurement of trabecular microarchitecture by evaluating pixel-grey-levels from lumbar spine DXA images [8]. Other quantitative methods have been introduced, such as quantitative computed tomography (QCT) and alternative non-ionizing methods, such as quantitative ultrasound (QUS) [9]. For QCT, a limitation is the lower availability and higher exposure to ionizing radiation, while QUS methods are limited by the fact that they are only applicable to peripheral sites like the heel, radius, tibia, and phalanx [10]. 

**Figure 1:**
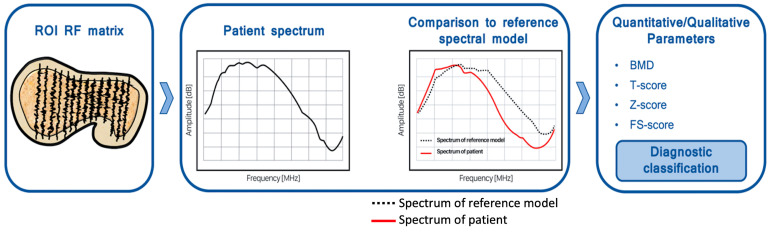
Examples of how REMS data deriving from femoral neck scans are analyzed to create a patient-specific spectrum, which is compared with spectral reference models. Regarding the spectra images, amplitude [dB] is on the y-axis, while frequency [MHz] is on the x-axis.

More recently, a new non-ionizing technique has been introduced with the name of radiofrequency echographic multi-spectrometry (REMS), which is based on a complete and complex spectral analysis of the raw, unfiltered ultrasound signals reflected from the bone surface of lumbar vertebrae and the femoral neck, the so-called radiofrequency (RF) ultrasound signals [11,12]. The main concept behind REMS technology is that “raw” ultrasound signals can provide useful information for bone status characterization. Usually, ultrasound devices for conventional imaging only use a small portion of the reflected ultrasound waves, filtering them out, to obtain a B-mode image [9,11,12]. Further, REMS can simultaneously analyze and acquire all RF wave spectra associated with the vertical lines of a given ultrasound image, laying the basis for the following statistical calculations needed to quantitative evaluate the examined bone [11,12,13]. Specifically, a REMS acquisition is obtained by placing the ultrasound probe on the abdomen or the hip to identify the target bone structure. The operator must set the scan depth to visualize the bone cortical interface in the central part of the sonographic field of view, with the transducer focus immediately above it. REMS software automatically detects the bone interface and identifies the region of interest (ROIs). For every sonographic line, a corresponding RF spectrum is extracted, and the software can identify cortical and trabecular bone layers. Moreover, the analysis of single scan line spectra allows the automatic exclusion of signal artifacts, such as those originating from osteophytes, thanks to the ability to detect “anomalous” characteristics in the frequency domain. Subsequently, the obtained spectrum deriving from the trabecular bone under investigation is analyzed to generate patient-specific spectra, which are compared to the previously derived anthropometrically matched reference models for pathological and normal bones, based on gender, age, BMI, and site [11,12]. Figure 1 shows an example of REMS acquisition and procession of RF signals at the femoral neck, as well as the comparison with spectral models. 

This procedure leads to the calculation of the so-called “Osteoporosis Score”, which corresponds to the percentage of analyzed spectra classified as “osteoporotic”. Employing linear equations, the Osteoporosis Score is then transformed into US-BMD values, expressed as g/cm^2^, and T-score and Z-score are calculated through quantitative comparisons with the National Health and Nutrition Examination Survey (NHANES) reference curves [11,12]. Several studies demonstrated a significant correlation between BMD measured by REMS and DXA [12,13,14,15]. Additionally, a growing body of evidence supports REMS as a diagnostic procedure that can be employed in evaluating patients with secondary osteoporosis. As an example, in 2022, Caffarelli et al. investigated the role of the REMS technique in the assessment of type 2 diabetes mellitus (T2DM), by comparing REMS to DXA at central sites in a cohort of 90 T2DM postmenopausal subjects matched with 90 healthy controls [15]. Compared to DXA, in which BMD measurements were higher in T2DM women, they discovered that REMS measurements were significantly lower in T2DM than in non-T2DM women. REMS was also able to classify more T2DM patients as “osteoporotic” compared to DXA, suggesting a promising role for this technology to complement DXA in assessing T2DM subjects [15]. Finally, in a subset of diabetic women with fragility fractures, the BMD values from both DXA and REMS at the lumbar spine were lower compared to subjects without fractures; nevertheless, the difference was statistically significant only for US-BMD by REMS [15]. Another interesting paper by Lalli et al. investigated the role of the Fragility Score (FS), which has been proposed as an indicator of bone quality derived by REMS [16]. In their paper, the authors assessed the discriminative power of FS in subjects with primary and disuse-related osteoporosis related to spinal cord injuries, showing a significant difference in the FS between the fractured and non-fractured subjects for both populations [16].

An important aspect of bone densitometry is the reproducibility of measurements, typically expressed as precision error and least significant change (LSC) [17]. Low precision error values are necessary to identify small BMD changes over time, which may be related to natural disease progression or therapeutic decisions [18]. In this context, it has been shown how increasing values of body mass index (BMI) can negatively influence the precision values of BMD at central sites [19]. Therefore, our study aims to determine the short-term intra-operator precision and short-term inter-operator repeatability of US-BMD by REMS at the lumbar spine (LS) and proximal femur at the level of the neck (FEM), exploring the effect of BMI increase on precision values.

## 2. Materials and Methods

### 2.1. Study Design

Our study was a single-center observational study. The assessment of short-term precision was conducted in a single center at the Galeazzi Hospital in Milan (Italy). The study protocol was approved by the Ethics Review Boards of the Galeazzi Hospital in Milan (protocol name: ULTRADXA; Comitato Etico San Raffaele, Milan, Italy). According to the International Society for Clinical Densitometry (ISCD) Official Positions, precision studies should be carried out on patients similar in age, gender, and bone density to those seen in daily clinical practice [20]. Therefore, we asked hospital staff women around the menopausal age (both pre- and post-menopausal) to voluntarily enter the study. According to our protocol, we included only women because they are the vast majority of subjects we usually test for BMD using DXA. Women voluntarily entered the study after providing written informed consent and authorization for anonymized data publication. All patients were recruited from November 2019 to February 2020. Inclusion criteria were Caucasian ethnicity, age between 30 and 80 years, and body mass index (BMI) below 35 kg/m^2^; exclusion criteria involved male gender and those conditions that could interfere with the REMS evaluation at the two skeletal sites (previous history of hip fracture or hip arthroplasty surgery, previous surgery at the lumbar spine). 

### 2.2. REMS Acquisitions

**Figure 2:**
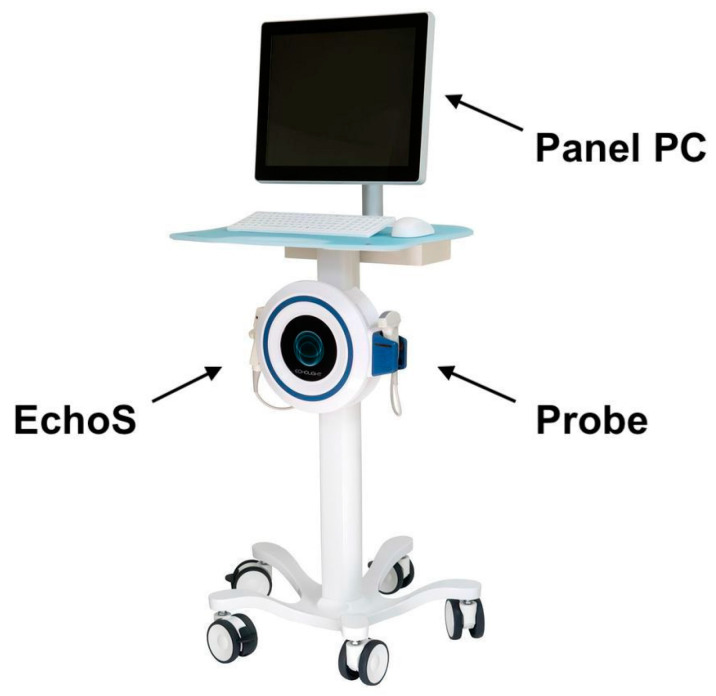
EchoStation device. Schematic representation of the EchoStation ultrasound machine, provided with the main unit EchoS, probe, and panel PC that implements REMS Technology.

All the enrolled patients underwent an ultrasound investigation of the spine and femur performed with the REMS technology for US-BMD determination. Two radiologists with 10 years (C.M.) and 7 years (S.G) in abdominal and musculoskeletal ultrasound performed all the acquisitions. A dedicated echographic device (EchoStation, Echolight Spa, Lecce, Italy) was employed to perform REMS scans as recommended by the manufacturer [11]. The system is equipped with a convex probe transducer operating at the nominal frequency of 3.5 MHz. Figure 2 shows the ultrasound device that was used for the study. 

For LS, the ultrasound transducer was placed under the sternum, to initially visualize L1 lumbar vertebra and then move downward until L4, according to the on-screen and audible indications provided by the device software. Each lumbar scan lasted 80 s (20 s per vertebra), followed by an automatic processing time of about 1–2 min. For FEM scans, the ultrasound transducer was placed parallel to the head-neck axis of the femur, to visualize the typical proximal femur cortical profile. The femoral neck scan lasted 40 s and was followed by an automatic processing time of about 1 min. 

For all the acquisitions performed at both axial sites, the operator firstly sets image depth and focus according to the patient’s constitution. Subsequently, after starting the scan, the algorithm automatically identifies the target bone interfaces in the ultrasound images and detects the region of interest (ROIs) for the following calculations. The bone interface is expected to fall within the ultrasound beam focal zone and at least 3 cm from the image bottom. 

To ensure maximum reliability of REMS diagnostic classifications, a rigorous quality check of the quality of all reports was performed according to a previous study [14]. Short-term intra-operator precision was calculated using data from two consecutive REMS acquisitions on the same patient by the same operator. Short-term inter-operator repeatability was obtained by performing a third REMS evaluation assessed by a second operator, on the same day. Both operators underwent specific training for using the EchoStation device and the REMS technology, which was performed on both phantoms and healthy volunteers (4 h for each operator). All consecutive REMS acquisitions at both axial sites were performed within the same day. 

**Figure 3:**
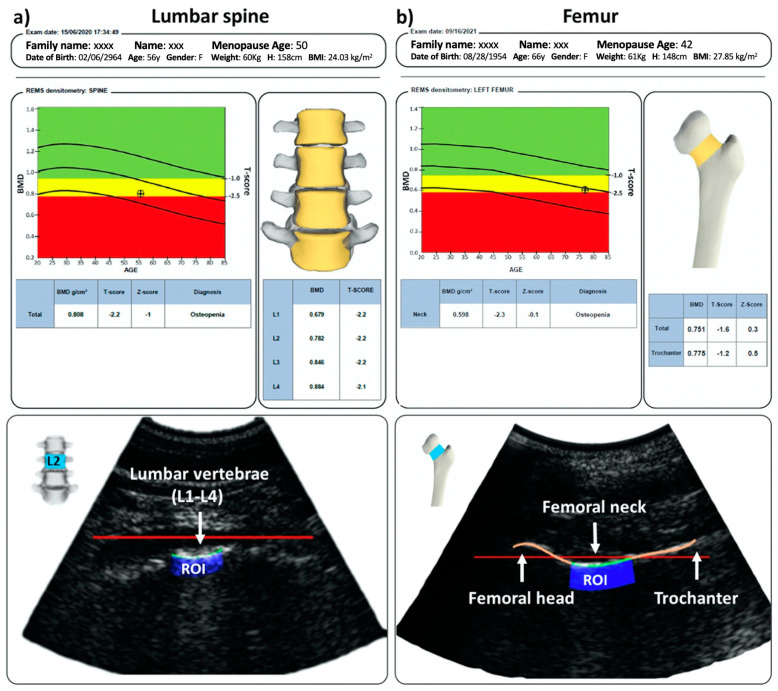
REMS acquisitions at the spine (**a**) and proximal femur (**b**). The figure depicts (upper panel) the medical report and (lower panel) a typical echographic image with the identification of the ROI (blue) and the bone interfaces (green). ROI, region of interest.

Figure 3 shows two representative images of subjects diagnosed with osteopenia, generated after a REMS scan performed at the spine (L1–L4), proximal femur (femoral neck, total femur), and the corresponding medical reports. The acquisitions display the bone interface, focus positioning, and the ROI on each axial site. 

### 2.3. Statistical Analysis

Similar to previous studies, precision error was obtained in compliance with the ISCD official position instructions, which suggest testing BMD on 15 patients 3 times, or 30 patients 2 times [20,21] to achieve statistical power. Two operators were involved in this study (CM and SG). The short-term intra-operator precision and inter-operator repeatability were calculated as the root-mean-square coefficient of variation (RMS-CV), the smallest detectable change (SDD), and least significant change (LSC) for a 95% confidence level, according to the ISCD official positions.

The participants were stratified based on their BMI resulting in two groups of <25 and >25 kg/m^2^, representing the optimal and overweight/obese groups, respectively. An unpaired t-test compared the US-BMD between the optimal weight and the overweight/obese groups. All calculations were performed using an Excel electronic sheet (Microsoft Excel 2019, Redmond, WA, USA), GraphPad Prism (v. 8.0.1), and MATLAB (v. R2013b). Data are presented as mean ± standard deviation (SD). The short-term intra-operator precision and inter-operator repeatability were calculated in these subgroups of patients also.

## 3. Results

**Figure 4:**
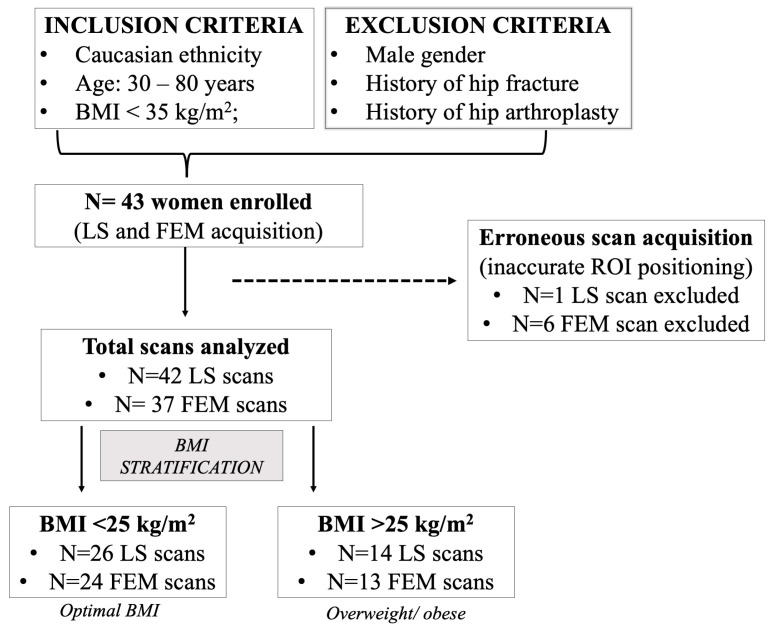
Study flowchart showing inclusion/exclusion criteria, the total number of women enrolled, the total scan analyzed after excluding erroneous acquisitions, and final BMI stratification. BMI = Body Mass Index; LS = lumbar spine; FEM = proximal femur; ROI = region of interest.

A total of 43 women were enrolled and underwent REMS scan both at the LS and the FEM site. Due to erroneous acquisition (inaccurate ROI positioning), one LS scan and six FEM scans were excluded; therefore, a total of n = 42 scans were analyzed at LS, while n = 37 scans were analyzed at FEM. For analysis convenience, the enrolled patients were divided into two groups so that the LS and FEM scans could be analyzed independently. A flowchart showing the inclusion/exclusion criteria, as well as the final number of scans analyzed according to the patient’s BMI, is reported in Figure 4. 

Erroneous acquisitions were mainly related to FEM scans and one case of LS scan, in which the operator did not correctly set image depth and focus. In such cases, the device cannot provide US-BMD data; therefore, the scans were excluded. Such erroneous acquisitions were obtained at the very early phase of our study, due to technical difficulties related to using a new machine. Still, they were easily overcome once the problem was understood.

The characteristics of the patients who received a REMS scan at the LS and FEM are shown in Table 1. All women were Caucasian, mostly postmenopausal, aged up to 66 years, with a mean age (±SD) of 48.9 ± 6.8 for LS and 48.3 ± 6.1 for FEM. Regardless of the site, the majority of women showed normal (38.1% at LS, 32.4% at FEM) or osteopenic (52.4% at LS, 62.2% at FEM) US-BMD values, while only a minority showed US-BMD values in the range of osteoporosis (9.5% at LS, 5.4% at FEM). 

Both intra- and inter-operator precision were assessed for the central axial sites (see Table 2). Regarding the intra-operator precision, RMS-CV was 0.47% for LS and 0.32% for FEM, with corresponding LSC values of 1.29% and 0.89%, respectively. Inter-operator repeatability error (RMS-CV) was 0.55% for the LS and 0.51% for the FEM, with corresponding LSC values of 1.52% and 1.40%, respectively.

To evaluate whether the precision was affected by the BMI variability among the enrolled subjects, the cohort (from the previous precision assessment study) was subdivided into two groups, in line with the WHO classification. Thus, patients with optimal weight (only one underweight patient was included in this group) were compared to overweight and obese subjects (see Table 3). Significant differences were found for the mean BMD between the normal and overweight/obese groups at both LS (*p* = 0.004) and FEM (*p* < 0.0001). The LSC values at the LS and FEM resulted in smaller in the optimal weight group with LSC = 1.23% and 0.73%, respectively, compared to the overweight/obese group with LSC = 1.40% and 1.07%, respectively.

## 4. Discussion

This study assessed the US-BMD precision at LS and FEM with REMS technology. Optimal values for intra-operator precision and inter-operator repeatability were found on both sites, which were superior to those typically documented with DXA when the characteristics of the study groups are analogous to those of the present study. The RMS-CV resulting from duplicate measurements at the spine by a single operator is 0.47% with REMS, whereas it is reported to range from 0.91% to 1.92% with DXA [22,23,24]. Similarly, the intra-operator repeatability expressed as RMS-CV at the femoral neck it is reported to range between 1.49% to 2.25% with DXA [24,25], while we found a smaller precision error of 0.32% with REMS. 

Inter-operator repeatability assessment studies using DXA are quite scant. To our knowledge, one study shared similar patient characteristics as this work and identified an RMS-CV value of 1.6% when measured with DXA at the spine [26], still superior to the value of 0.55% found here by applying REMS technology. 

In addition, the measurement error of DXA scanners is also affected by other factors such as inter-device variability [27], patient positioning, and post-acquisition analysis errors [7,28]. Considering the possible negative effect on the precision with DXA related to patient positioning, the margin of error with DXA could still be large [29]. On the contrary, it has been shown that REMS analysis is independent of patient positioning and inter-device variability [30].

The evaluation of the precision error of any densitometric device is fundamental in the clinical practice to warrant that over a certain time-lapse, any detected skeletal change is due to a bone density variation and not to the instrument’s uncertainty nor the operator’s experience [31]. In clinical practice, a BMD change that overcomes the precision error of a given densitometer is attributed to a meaningful biologic variation, identified with the LSC value. Therefore, minimizing precision errors ensures that the test is sensitive and accurate enough to detect subtle skeletal changes, thus enabling thorough monitoring of bone health and selecting the most reasonable therapeutic option for the patient [32]. 

Generally, when performing precision studies, cohort characteristics should be carefully selected since factors such as body mass index (BMI), study group as well as ethnicity, may impact the precision assessment. Regarding BMI-associated errors, a tendency for precision errors and variability of serial measurements to increase with increasing fat layering has been frequently observed in obese patients when using DXA [19,33], probably due to a reduction in X-ray penetration into thicker soft tissues [19]. 

On the contrary, when the cohort was stratified in two distinct BMI groups, REMS provided considerably high precision and repeatability, which were superior in subjects with normal weight. Indeed, considering that REMS can automatically identify the bone interface and then analyze the ultrasonic signal reflected by the bone region of interest in a highly selective way, it may still have a superior ability concerning DXA in dealing with physical interferences induced by fat tissues. For instance, during a REMS scan at the lumbar spine, the attenuation of the signal caused by the encumbrance of abdominal fat can be counteracted by increasing the focus and depth values and pressing the transducer on the abdomen, thus maximizing penetration of the ultrasonic beam on the bone interface [34]. On the other hand, DXA is a technique that measures the areal bone density from a bi-dimensional image obtained as a planar projection of the scanned tissues, without considering the effects of different volumetric tissues above the ROI. In fact, among others, this technique greatly suffers from the contribution of interposed soft tissues contained in the scan, which are projected on the image modifying the pixel values in the ROI and consequently influencing the bone density estimation [35]. 

Regarding the subjects investigated, for instance, postmenopausal women with osteoarthritis may have greater BMD variability compared to healthy subjects [36]. Amorim and colleagues have recently reported inter-operator repeatability errors with REMS to be above 1% at both femoral neck and lumbar spine, overall resulting in a slightly lower precision compared to the data previously published [14] as well as to the current work. Nevertheless, this work included a multiracial study group of Asian, Caucasian, and African descendant women, also affected by degenerative disorders. Therefore, it could be speculated that in a multiracial real-life group also, REMS precision performance is very good. 

REMS analysis depends on the automatic processing of unfiltered native ultrasound signal and subsequent comparison of its spectral profile to a dedicated database of spectral models for healthy and osteoporotic bones to assess the diagnostic classification. Bone mass modulates the ultrasound echo and the associated spectra as a function of its physical properties [11,12]. After discarding artifacts, unfiltered signals of multiple scan lines are processed in parallel to obtain a patient-specific spectrum profile.

Considering that in the clinical routine, REMS scans can be repeated as often as needed, thanks to their non-invasive nature, it overcomes certain limitations of the DXA acquisition. Although DXA images can undergo several post-processing analyses, the initial quality of the scan must comply with high ISCD quality standards to avoid the detected BMD measurement being misleading. Furthermore, the REMS method can reduce reproducibility errors by automatically verifying whether the spectral features of the tested area correspond with the spectral model of the trabecular bone. If the ROI is considered non-diagnostic or the signals provided are of insufficient quality, the operator does not receive the test result and will need to perform the test again [11,12]. At the same time, a rigid training operator program for using REMS is needed to assure maximum diagnostic accuracy.

This study has some limitations, mainly related to the limited sample size that did not allow us to perform a complete stratification according to the various categories of BMI. Nevertheless, we reach a statistical power for the purpose of precision assessment, and further studies will explore the effect of soft tissue on US-BMD in very obese patients. Another possible limitation as a source of bias relates to the study population, which was entirely composed of Caucasian women. Finally, as our study was focused on the precision assessment of US-BMD values, we can neither provide data nor draw conclusions regarding the accuracy of the REMS technique. Nevertheless, our paper was specifically aimed at assessing the technical aspect of REMS precision according to BMI. Previous studies assessed the REMS accuracy compared to DXA on large populations, showing good agreement between US-BMD and the corresponding DXA-measured BMD [14,37]. 

In conclusion, US-BMD assessed with REMS technology can be used to precisely monitor bone density. Further studies will help define the proper interval time for longitudinal assessment of US-BMD with REMS.

## Figures and Tables

**Table 1:** Characteristics of the study group. Data are presented as mean ± SD of the total number of acquisitions performed at the respective axial sites (LS and FEM). BMD, bone mineral density; BMI, body mass index; FEM, femoral neck; LS, lumbar spine; SD, standard deviation. Regarding diagnosis, percentages are in parentheses.

Demographic Data	LS (42)	FEM (37)
Age (years)	48.9 ± 6.8	48.3 ± 6.1
Ethnicity	Caucasian	Caucasian
Diagnosis		
*Normal*	16 (38.1%)	12 (32.4%)
*Osteopenia*	22 (52.4%)	23 (62.2%)
*Osteoporosis*	4 (9.5%)	2 (5.4%)
BMI (kg/m^2^)	24.71 ± 4.21	25.0 ± 4.84
BMD (g/cm^2^)	0.914 ± 0.1	0.709 ± 0.1
Age (years)	48.9 ± 6.8	48.3 ± 6.1

**Table 2:** Assessment of short-term precision. CV, coefficient of variation; FEM, femoral neck; LS, lumbar spine; LSC, least significant change; RMS, root mean square.

Short Term Precision						
	LS (42)			FEM (37)		
	RMS-CV (%)	LSC (%)	SDD (g/cm^2^)	RMS-CV (%)	LSC (%)	SDD (g/cm^2^)
Intra-operator precision	0.47	1.29	0.009	0.32	0.89	0.004
Inter-operator repeatability	0.55	1.52	0.009	0.51	1.40	0.008

**Table 3:** Precision error in subjects with optimal weight or overweight/obese patients at the LS and FEM. A *t*-test was used to determine significant differences between BMD values measured the optimal weight and overweight/obese groups at the LS (*p* = 0.004) and FEM (*p* < 0.0001). BMD, bone mineral density; BMI, body mass index; CV, coefficient of variation; FEM, femoral neck; LSC, least significant change; LS, lumbar spine; RMS, root mean square; SDD, smallest detectable difference. Optimal category = BMI ≤ 25 kg/m^2^; Overweight/obese category = BMI > 25 kg/m^2^.

Site	BMI Category	*n*	BMD (g/cm^2^)	*p*-Value	RMS-CV (%)	LSC (%)	SDD (g/cm^2^)
***LS***	Optimal ^$^	26	0.879 ± 0.10	*p* = 0.004	0.44	1.23	0.008
	Overweight/obese	14	0.975 ± 0.09		0.50	1.40	0.011
***FEM***	Optimal ^$^	24	0.659 ± 0.07	*p* < 0.0001	0.28	0.73	0.004
	Overweight/obese	13	0.794 ± 0.09		0.39	1.07	0.005

^$^ (only one underweight patient was included in the normal group).

## Data Availability

Not applicable.
